# A new environmentally resistant cell type from *Dictyostelium*

**DOI:** 10.1099/mic.0.2006/000562-0

**Published:** 2007-02

**Authors:** Ioannis Serafimidis, Gareth Bloomfield, Jason Skelton, Al Ivens, Robert R. Kay

**Affiliations:** 1 MRC Laboratory of Molecular Biology, Hills Road, Cambridge CB2 2QH, UK; 2 The Wellcome Trust Sanger Institute, Hinxton, Cambridgeshire CB10 1SA, UK

## Abstract

This paper describes the serendipitous discovery and first characterization of a new resistant cell type from *Dictyostelium*, for which the name aspidocyte (from *aspis*: Greek for shield) is proposed. These cells are induced from amoebae by a range of toxins including heavy metals and antibiotics, and were first detected by their striking resistance to detergent lysis. Aspidocytes are separate, rounded or irregular-shaped cells, which are immotile but remain fully viable; once the toxic stress is removed, they revert to amoeboid cells within an hour. Induction takes a few hours and is completely blocked by the protein synthesis inhibitor cycloheximide. Aspidocytes lack a cell wall and their resistance to detergent lysis is active, requiring continued energy metabolism, and may be assisted by a complete cessation of endocytosis, as measured by uptake of the dye FM1-43. Microarray analysis shows that aspidocytes have a distinct pattern of gene expression, with a number of genes up-regulated that are predicted to be involved in lipid metabolism. Aspidocytes were initially detected in a hypersensitive mutant, in which the AMP deaminase gene is disrupted, suggesting that the inductive pathway involves AMP levels or metabolism. Since aspidocytes can also be induced from wild-type cells and are much more resistant than amoebae to a membrane-disrupting antibiotic, it is possible that they are an adaptation allowing *Dictyostelium* cells to survive a sudden onslaught of toxins in the wild.

## INTRODUCTION

The success of micro-organisms in resisting environmental toxins and antibiotics may be a major determinant of their overall success. For free-living organisms this resistance would be important in competition for ecological niches, and for pathogens in blunting the effect of antibiotic treatment. Strategies employed by bacteria include the expression of resistance genes, the formation of dormant spores (Errington, 2003) or protected biofilms (Hall-Stoodley *et al.*, 2004; Hoffman *et al.*, 2005) and the maintenance of a small population of slow-growing ‘persister’ cells, which can survive a sudden antibiotic onslaught (Balaban *et al.*, 2004).

*Dictyostelium* amoebae probably face a similar challenge in the wild, since their natural habitat of soil contains a variety of antibiotic-producing bacteria and fungi and may also suffer from contamination with heavy metals and chemical residues. Social amoebae also compete with each other for resources (Horn, 1971; Ketcham *et al.*, 1988), and their recently appreciated capacity for making polyketides (Austin *et al.*, 2006; Eichinger *et al.*, 2005; Serafimidis & Kay, 2005) suggests that some of this competition is mediated by amoebocidal chemicals (Horn, 1971) and defences against these chemicals.

The *Dictyostelium discoideum* genome reveals a wide array of potential resistance genes (Burlando *et al.*, 2002; Eichinger *et al.*, 2005) and this organism can also form physically resistant macrocysts and spores, encased in cellulosic coats (Kessin, 2001; Raper, 1984). However, macrocysts and spores only differentiate from starving cells, and both require the cooperation of hundreds to thousands of cells and around 24 h to form. Their major ecological function appears to be long-term survival and dispersal (and sexual recombination for macrocysts) rather than a short-term protection against toxins (Huss, 1989; Wallace & Raper, 1979). Microcysts are known from other species of social amoeba, but have not been reported from *D. discoideum* (Raper, 1984). The naked amoebae of this species are therefore a somewhat vulnerable state, for which some means of protection against chemicals and antibiotics would be advantageous.

The discovery is reported here of a new resistant cell type, the aspidocyte, by whose formation both growing and starving *Dictyostelium* cells may defend themselves against toxic attack.

## METHODS

### Organisms, media and growth conditions.

*D. discoideum* Ax2 (Watts & Ashworth, 1970) was used as the wild-type strain in most experiments; the construction of mutant strains is described below. Strains were grown in axenic medium (Watts & Ashworth, 1970) supplemented with vitamins (0.1 mg cycancobalamin l^−1^, 0.02 mg biotin l^−1^, 0.2 mg riboflavin l^−1^) or in association with *Klebsiella aerogenes* (*K. pneumoniae*) on SM-agar plates (Kay, 1987). Growth and development were at 22 °C. *Dictyostelium firmibasis* and *Dictyostelium mucoroides* were a kind gift from Dr Pauline Schaap, University of Dundee, UK.

### Aspidocyte induction.

In the standard method, cells were washed free of growth medium or bacteria by low-speed centrifugation from KK2 (16.5 mM KH_2_PO_4_, 3.9 mM K_2_HPO_4_, 2 mM MgSO_4_, pH 6.1) and resuspended at 10^6^ ml^−1^ in spore medium: 10 mM MES, 20 mM KCl, 20 mM NaCl, 1 mM CaCl_2_, 1 mM MgCl_2_, pH 6.2, with 100 μg streptomycin ml^−1^ and distributed into tissue culture dishes (1.5 ml per 3.5 cm diameter dish). After 1 h, 2 or 5 mM cAMP and the compounds indicated in the text were added and incubation continued for 12–18 h, or as indicated in the text. Aspidocytes were scored following lysis of amoebae with 0.3 % or 0.2 % Cemulsol NP12 (Rhone Poulenc) for at least 4 min, either by phase-contrast microscopy, or by serial dilution onto SM-agar plates with bacteria, and subsequent plaque counting.

Aspidocytes were produced for microarray analysis using HM1158 (*amdA*) cells (see below) grown in axenic medium with phosphate buffer replaced by 20 mM MES pH 6.2 since phosphate is inimical to aspidocyte formation, and in experiments not shown, aspidocytes were also induced in axenic medium. After harvesting and washing, cells were resuspended at 5×10^6^ ml^−1^ in spore medium, shaken at 180 r.p.m. and aspidocytes induced for 6 h with either 25 μM CdCl_2_/10 μM diethylstilboestrol (DES) (five biological replicates, 67 % induction) or 250 μM thiabendazole (two biological replicates, 49 % induction). Each biological replicate was accompanied by an uninduced control sample.

For electron microscopy, aspidocytes and control cells were produced in the standard conditions and fixed with 1 % glutaraldehyde in 20 mM MES, 10 mM NaCl, 1 mM MgCl_2_, 1 mM CaCl_2_, pH 6.2 for 3 h on ice, followed by osmication and preparation for electron microscopy by standard methods, all performed by Dr Jeremy Skepper, University of Cambridge.

Confocal images and time-lapse films were taken using a Bio-Rad Radiance system mounted on a Nikon inverted microscope.

### Molecular biology.

REMI mutagenesis (Kuspa & Loomis, 1992) of Ax2 cells used pBRS1 vector and DpnII introduced by electroporation. A pooled library of ∼180 000 independent transformants, selected with 10 μg Blastocidin ml^−1^, was subjected to three rounds of selection for mutants insensitive to the endogenous stalk inducer DIF. Cells at 10^6^ ml^−1^ in spore medium plus 15 mM 8-Br-cAMP, 25 μM CdCl_2_ and 100 nM DIF-1 [1-(3,5-dichloro-2,6-dihydroxy-4-methoxyphenyl)-1-hexanone] (10 ml per 9 cm diameter dish) were incubated for 20 h, then 0.3 % Cemulsol NP12 was added to lyse amoebae. Surviving spores (and aspidocytes) were spun down and grown up in bulk on bacteria for the next round of selection, or as clones, for examination. The site of REMI insertion was identified in strain HM556 as the *Dpn*II restriction site 57 bases downstream of ATG of the coding sequence by inverse PCR (Keim *et al.*, 2004) and DNA sequencing. A knockout vector, pIS11, was made from this strain by plasmid rescue following *Hin*dIII digestion of the nuclear DNA, and used to disrupt the gene in Ax2, creating strain HM1158. Strain HM1158 was transformed with the ABD-GFP reporter (Pang *et al.*, 1998) by standard methods.

### Microarray analysis.

Total RNA was extracted using RNeasy kits (Qiagen) and 25 μg RNA per sample per microarray labelled by direct incorporation of Cy3- or Cy5-conjugated dCTP (Amersham) using reverse transcriptase (Superscript III, Invitrogen). Samples were paired and hybridized on custom microarrays, printed with ∼9300 features in duplicate (http://www.sanger.ac.uk/PostGenomics/PathogenArrays/Dicty/protocols/). Five independent replicates of aspidocytes induced with CdCl_2_/ DES were compared directly with uninduced controls. Three of these replicates contributed one array each (two replicates in one dye-orientation, one in the other), while two contributed two arrays (of both dye-orientations), making seven arrays in total. The latter two replicates also formed part of a larger design comparing (**A**) control cells with (**B**) CdCl_2_/DES-induced aspidocytes, (**C**) thiabendazole-induced aspidocytes, and (**D**) the starting population of vegetative cells. Samples were compared in a loop of **A** with **B**, **B** with **C**, **C** with **D**, and **D** with **A**. Two independent replicates were carried out, each in both dye-orientations, giving a total of 16 arrays for this experiment.

Arrays were scanned using an Axon Instruments Genepix 4000B scanner and the resulting images quantified (Genepix 3.0, Axon Instruments) and analysed using the Bioconductor package Limma (Gentleman *et al.*, 2004; Smyth, 2004, 2005). Background fluorescence was subtracted (Kooperberg *et al.*, 2002), and the resulting log-ratios normalized by the print-tip loess function of Limma. In the larger design above, expression levels for each treatment were related back to uninduced cells in axenic medium by linear model fitting (using generalized least squares) and comparisons of interest then inferred. The significance of apparent differential expression was assessed by a Bayesian approach (Smyth, 2004), controlling the false discovery rate (Benjamini & Hochberg, 1995) at 5 %. Gene identifiers given in the text refer to the annotated genome accessible at GeneDB (http://www.genedb.org/genedb/dicty/index.jsp) and Dictybase (http://dictybase.org/).

The dendrogram was based on the Spearman correlations of relative expression values for genes significantly different from starting vegetative cells in any other of the conditions. This filtering step was used to exclude noisy data from the analysis. The function spearman.dist in the Bioconductor package bioDist (B. Ding, R. Gentleman & V. Carey – bioDist: Different distance measures. R package version 1.2.0) was used to convert the correlation scores into a distance metric, which was used to generate a tree using phylip (Felsenstein, 2005).

The heatmap was generated using the heatmap.2 function of the R package gplots [Gregory R. Warnes. Includes R source code and/or documentation contributed by Ben Bolker and Thomas Lumley (2005). gplots: Various R programming tools for plotting data. R package version 2.1.1] using the euclidean distances between expression values and the ‘complete’ agglomeration method to order the genes.

Quantitative real-time RT-PCR was carried out using a Stratagene Mx3000P instrument and Invitrogen Superscript III Platinum SYBR Green One-Step qRT-PCR kit, according to the manufacturer's instructions, with the exception that reverse transcription was prolonged to 20 min. PCR cycling conditions were 95 °C for 10 min followed by 40 cycles of 94 °C for 30 s, 52 or 58 °C (depending on primers) for 45 s, 68 °C for 45 s. After this, products were briefly melted, annealed and remelted to obtain a dissociation curve. Only primers that resulted in a single peak were accepted. The threshold cycle (Ct) for each amplification was determined by the amplification-based threshold method, and baseline corrected by the adaptive baseline method of the Stratagene Mx3000P software. Primers (forward, reverse): DDB0167376 GCTATGACCCCCAAGGATTCACAAAATACAGC and GCTCTACAGGTCCGATATCATTCTCTTCGACT; DDB0167446 AGATTTTAGGTTGTCTTGATTCAG and AGAGATTGTAGAAATGACTTTTGC; DDB0216948 GTAGCTGGTAAACAATCAGAAACT and AGATAGGATTTGAAAAATCAGTTG; DDB0190107 GCTATGACCCCCGAATTTGGTTCCTGTAAAAA and GCTCTACAGGTCATTTACCTGGGAATACACAA; DDB0217770 GCTATGACCCCCTCAAGTGTTATCAAATGCAC and GCTCTACAGGTCTCCTCTGAAAGATCCAATAG; DDB0217855 GCTATGACCCCCCAAATCATTCTCTCCCTCTA and GCTCTACAGGTCTTACTACGGAAAAGATCACC; DDB0206063 GCTATGACCCCCGTCTTAAAGGAAGGTGTTGA and GCTCTACAGGTCTACTGGACGATAGTTAAGGG.

For each gene of interest a standard curve of Cts for four quantities of total RNA was produced in duplicate and a linear model fitted using the lm() function in R. Cts for two different quantities of both control RNA (cells starved in spore medium) and aspidocyte RNA (cells treated with Cd^2+^ and DES) in duplicate were read off the standard curve, giving four measurements of starting mRNA concentration for each gene in each RNA. The geometric mean ratio between aspidocyte and control RNA samples for each gene of interest was normalized by subtracting the geometric mean of ratios obtained similarly for three reference genes (for which there was little evidence of differential expression in the array data – Dictybase IDs: DDB0167376, DDB0217770, DDB0206063).

## RESULTS

### Discovery of aspidocytes

Aspidocytes were discovered by chance in a screen for mutants that cannot respond to DIF-1 (Morris *et al.*, 1987), a signal molecule that induces stalk cell differentiation during *Dictyostelium* development. The screen was based on the ability of DIF-1 to suppress the alternative fate of spore formation in monolayer cultures (Kay, 1989), and hence unresponsive mutants were sought as those still able to form spores in the presence of DIF-1. Spores were selected at the end of the induction by adding a non-ionic detergent to lyse any remaining amoebae, stalk cells being already dead. We varied from previous work (Thompson *et al.*, 2004) by adding a low concentration of Cd^2+^ ions to the culture to increase the overall efficiency of cell differentiation (Serafimidis & Kay, 2005), but by happenstance this led instead to the isolation of a mutant that forms aspidocytes (see below).

**Fig. 1. f1:**
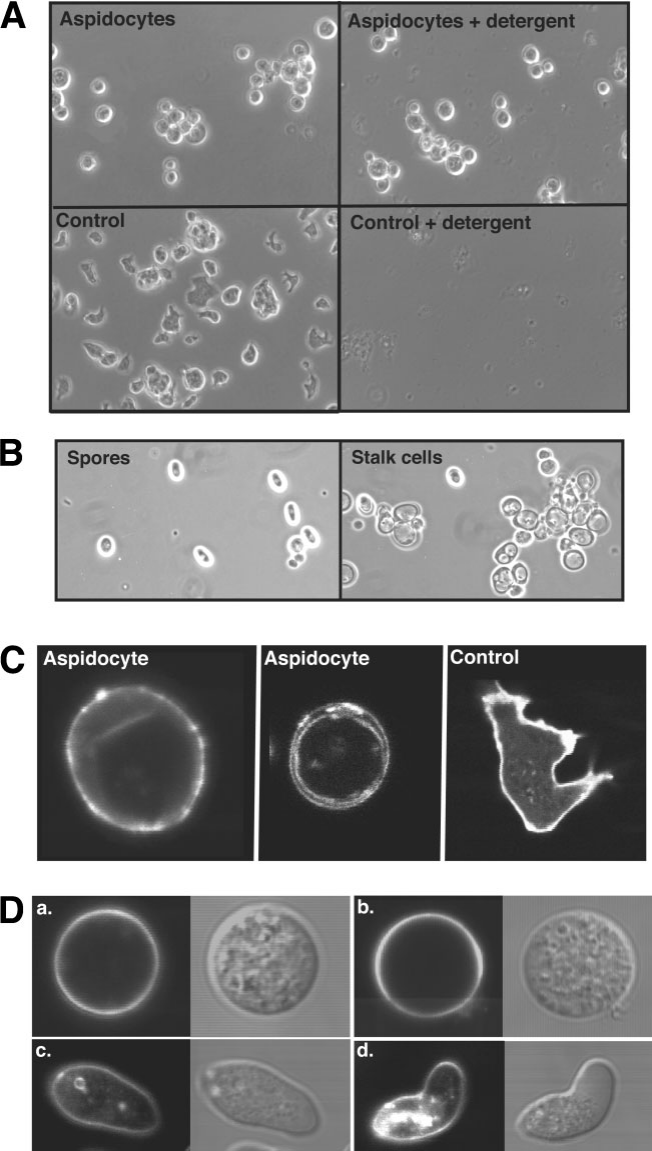
Detergent resistance of aspidocytes, their actin cytoskeleton and endocytosis. (A) Aspidocytes are resistant to detergent lysis. Aspidocytes were induced from strain HM1158 (*amdA*) in the standard conditions, using 25 μM CdCl_2_ and 100 nM DIF-1, or lacking CdCl_2_ for the control. After 12 h, 0.3 % Cemulsol NP12 was added and the plates photographed after another 20 min. Control amoebae have lysed, but the aspidocytes remain. Phase-contrast images. (B) For comparison, spore and stalk cells induced in monolayer culture from strain V12M2 by 8-Br-cAMP and 100 nM DIF-1 (for stalk cells; see Kay, 1989). (C) F-actin distribution in aspidocyte and control cells expressing ABD-GFP, which binds to F-actin. Confocal images. (D) Endocytosis by aspidocytes and control cells detected using the fluorescent dye FM1-43. Pairs of fluorescent confocal and differential interference contrast (DIC) images are shown of aspidocytes (a, b) or control cells (c, d), either immediately after staining (a, c) or after 20 min incubation with 2 μM FM1-43 (b, d). This dye is only fluorescent when inserted into the membrane, and only enters the cell by endocytosis.

After three rounds of induction with DIF-1 followed by detergent selection, an unusual mutant was obtained from a library of insertional mutants. Cells of this mutant readily survived a detergent selection (0.3 % Cemulsol NP12), but they did not form spores as expected, nor did they resemble vacuolated stalk cells: instead rounded, or irregular-shaped cells remained (Fig. 1A, B[Fig f1]). Because of their resistance to detergent lysis, we named these cells aspidocytes (from *aspis*: Greek for shield).

As the original aspidocyte-forming strain was created by REMI mutagenesis (Kuspa & Loomis, 1992), the affected locus should be marked by the insertion of the REMI plasmid. We therefore identified this locus by rescue and sequencing of the flanking DNA (Keim *et al.*, 2004) and found that that the disrupted gene (*amdA*) encoded the enzyme AMP deaminase. To confirm that the mutant phenotype is due to disruption of *amdA*, we redisrupted this gene by homologous recombination in the wild-type Ax2 background, giving strain HM1158, and also obtained a disruption in the Ax4 background from another laboratory (Chae *et al.*, 2002). Both strains formed aspidocytes as readily as the original mutant (not shown) and as these strains were so similar, only HM1158 was used in subsequent experiments. Development of these strains is not normal: they produce small fruiting bodies and far fewer spores than the wild-type, as has already been described (Chae *et al.*, 2002).

### Aspidocyte inducers and induction of the wild-type

To understand the conditions necessary to induce aspidocytes, we first varied single components from the original medium in which they formed. This medium contained 8-Br-cAMP, which could be replaced by cAMP without affecting aspidocyte yield, thus giving the complete medium shown in Table 1. In this medium about 70 % of HM1158 amoebae were converted to aspidocytes. For each condition aspidocytes remaining after detergent treatment were scored both by phase-contrast microscopy and by plating them clonally with bacteria for viability. Omission of components in turn from the medium showed that the key aspidocyte inducer is cadmium, and that DIF-1 acts synergistically with it (Table 1). cAMP has only a minor impact but was usually included in assays for convenience as its presence inhibits aggregation of the starving cells. The concentrations of Mg^2+^, Ca^2+^, K^+^ and Na^+^ were then independently varied and aspidocytes found to form quite efficiently in a wide range of ionic conditions, including the complete omission of divalent cations (not shown). The conditions of Table 1 are already close to optimal and were therefore retained.

A variety of potential aspidocyte inducers was then tested over a range of concentrations, both alone and in the presence of a low concentration of either DIF-1 or Cd^2+^ ions, to detect possible synergistic interactions (Table 2). Aspidocytes were scored as detergent-resistant cells and given as a percentage of input cells for convenience.

Inducers include: the heavy metal ions Cd^2+^ and Hg^2+^ (Co^2+^, Mn^2+^, Ni^2+^ and Zn^2+^ were much less effective or inactive at 0.2 mM; not shown); thiabendazole, a mitotic inhibitor in *Dictyostelium* (Welker & Williams, 1980); a group of azole anti-fungal agents that block sterol synthesis, represented by clotrimazole and econazole (Lupetti *et al.*, 2002); sublethal doses of cycloheximide (protein synthesis) and actinomycin D (RNA synthesis) and G418, the aminoglycoside antibiotic widely used as a selective agent in *Dictyostelium* transformation (Nellen *et al.*, 1984). In addition, the non-physiological stalk cell inducers DES, miconazole and zearalenone are strongly active and may work through inhibition of the plasma membrane proton pump (Gross *et al.*, 1983, 1988; Kay *et al.*, 1986; Pogge-von Strandmann *et al.*, 1984).

Synergy is clearly evident between some combinations of inducers, such as 25 μM CdCl_2_ and 100 nM DIF-1, where summing their individual potencies would predict that the two compounds together would give less than 3 % aspidocytes, whereas in fact they give 66 % aspidocytes. DIF-1 is also strongly synergistic with low concentrations of cycloheximide, actinomycin D and G418, while 25 μM CdCl_2_ is only strongly synergistic with thiabendazole.

DIF-1 could be efficiently substituted in synergy experiments by the closely related DIF-2 and DIF-3, which also induce stalk cell differentiation (Masento *et al.*, 1988), but not so well by its biosynthetic precursors THPH, Cl-THPH and dM-DIF-1 (Kay, 1998) which have greatly reduced stalk-cell-inducing activity (Table 3).

Aspidocytes can also be induced in conditions suitable for normal development: starving HM1158 cells plated on agar containing either 10 μM miconazole or 300 μM thiabendazole remain as an even lawn and after 24 h have formed more than 50 % aspidocytes (not shown). Thus it appears that aspidocyte formation can override normal development.

Initially aspidocytes could only be efficiently induced in the *amdA* mutant strains, and the parental strain Ax2 gave less than 10 % induction (Table 1). However, a combination of 25 μM Cd^2+^ and 10 μM DES worked well for starving Ax2 cells, giving 69±4.9 % aspidocytes in the conditions of Table 1, and also allowed 10 % aspidocyte induction in growth medium with the *amdA* mutant.

Aspidocytes, again defined as cells resistant to 0.3 % Cemulsol NP12, can also be induced by this combination from starving cells of the wild-type isolates NC4 and V12M2 of *D. discoideum*, and from the related species *D. firmibasis* and *D. mucoroides*. All these strains produced approximately 5 % aspidocytes, by viability after detergent treatment, using 50 μM CdCl_2_ and 10 μM DES in the conditions of Table 1 (not shown).

### Aspidocyte differentiation

**Fig. 2. f2:**
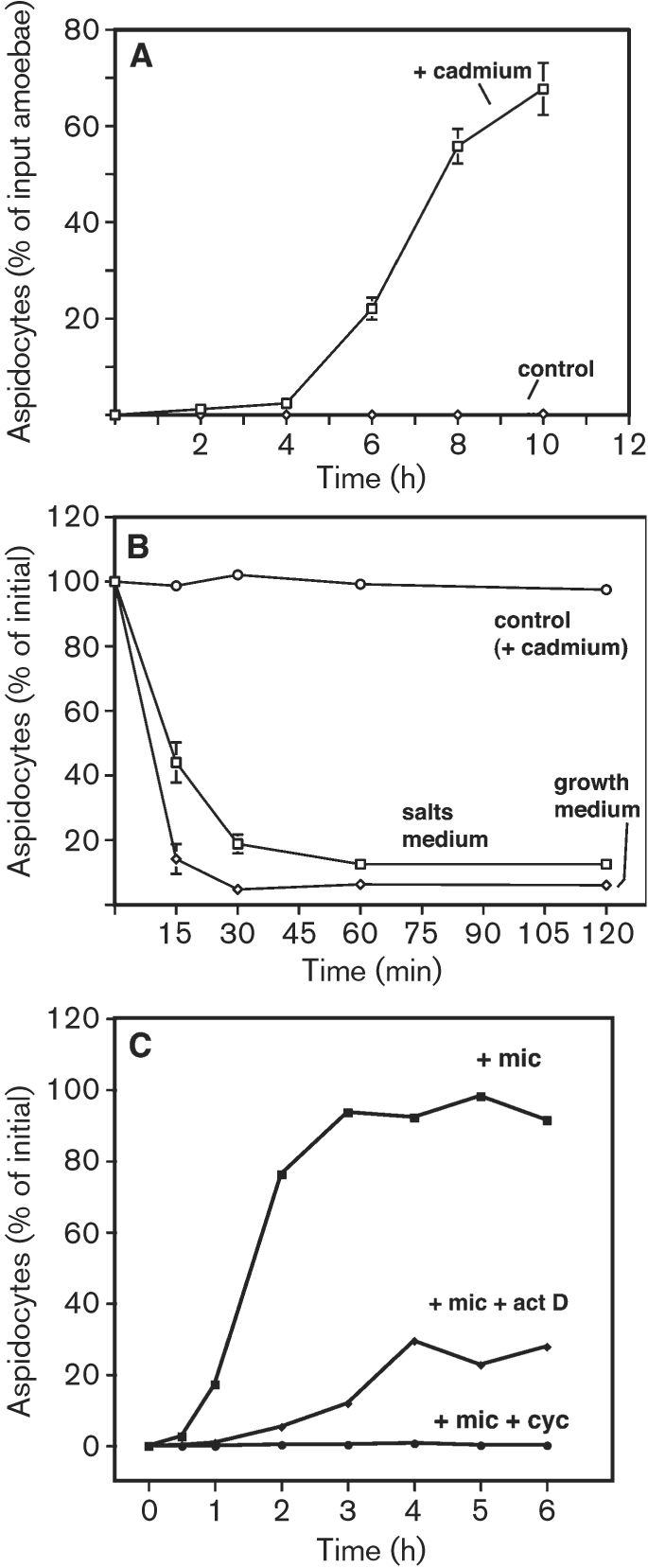
Kinetics of aspidocyte formation and dissolution. (A) Kinetics of aspidocyte formation. Control cells have no Cd^2+^. (B) Kinetics of aspidocyte dissolution. (C) Inhibition of aspidocyte formation by cycloheximide and actinomycin D. Aspidocytes were induced in strain HM1158 in the standard conditions and determined by counting after detergent lysis. In (B), aspidocytes induced after 12 h were washed, and resuspended either in the same medium, with or without 25 μM Cd^2+^, or in axenic medium without Cd^2+^. In (C), the additions were: 10 μM miconazole (mic), 1.8 mM cycloheximide (cyc) and 100 μM actinomycin D (act D) as indicated. Note that miconazole induces aspidocytes much more quickly than CdCl_2_/DES (compare panels A and C). A and B are from three and C from two independent experiments.

Aspidocyte formation takes one to several hours depending on the inducer (Fig. 2A, C[Fig f2]). Time-lapse filming shows that the amoebae move actively for a while, but then gradually cease moving and forming pseudopodia, though cytoplasmic movements can still be seen. These cells remain fully viable, and if the inducers are washed away, they once again become detergent-sensitive (Fig. 2B[Fig f2]) and many of them resume amoeboid movement within 1 h (see movies 1 and 2, available as supplementary data with the online version of this paper).

The slow evolution of detergent resistance as aspidocytes form contrasts with the rapid response of *Dictyostelium* cells to certain other stresses such as osmotic shock (Zischka *et al.*, 1999) and suggests that changes in gene expression may be involved in aspidocyte formation. As a first test of this idea, we asked whether inhibitors of transcription and translation inhibit aspidocyte formation. Cycloheximide strongly inhibits protein synthesis in early development at the concentration used in Fig. 2C[Fig f2], but is not immediately lethal, as cells can recover even after 24 h of treatment; actinomycin D inhibits transcription, but mRNA production is only incompletely inhibited (Firtel *et al.*, 1973; Mizukami & Iwabuchi, 1970). Fig. 2C[Fig f2] shows that aspidocyte production is completely inhibited by cycloheximide and partially inhibited by actinomycin D, consistent with an essential requirement for protein synthesis and an important one for RNA synthesis.

We next used micro-arrays carrying 9300 probes to compare expression profiles of aspidocytes with their uninduced controls and with the starting vegetative cells. Induction was performed in suspension for 6 h without cyclic-AMP using either thiabendazole or CdCl_2_/DES.

**Fig. 3. f3:**
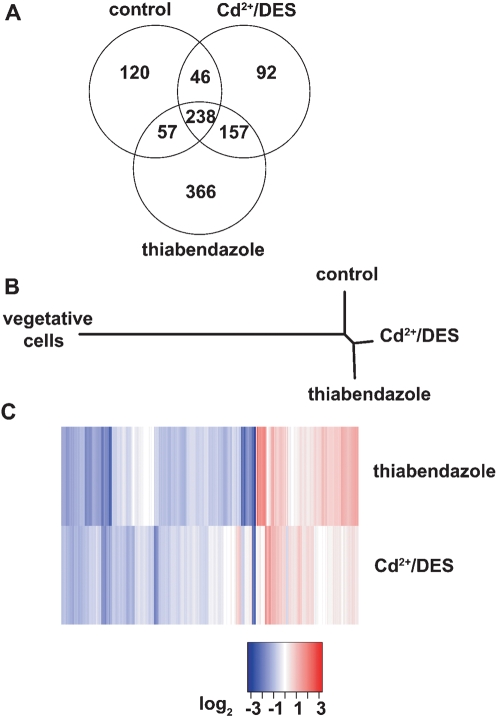
Micro-array analysis of aspidocyte gene expression. (A) Venn diagram of genes significantly (*P*<0.05) up-regulated in aspidocytes and control cells, compared to vegetative amoebae. (B) Phylogram based on genes expressed significantly differently from vegetative amoebae in any of the other conditions. (C) Heatmap comparing 953 genes significantly up- or down-regulated in aspidocytes compared to uninduced control cells (FDR-adjusted F.p.value <0.05). Aspidocytes were induced in starving suspensions of strain HM1158 with either 25 μM Cd^2+^/10 μM DES or 250 μM thiabendazole for 6 h (two independent replicates) and gene expression patterns determined using a ∼9300 feature custom micro-array, as described in Methods. Genes up-regulated in aspidocytes compared to their controls are in red and those down-regulated are in blue. The colour scale is log_2_.

Fig. 3A[Fig f3] shows a Venn diagram of all the genes significantly (*P*<0.05) up- or down-regulated in control and thiabendazole- or CdCl_2_/DES-treated cells, compared to vegetative amoebae. Overall, the expression of 1076 genes changes significantly in one or more condition, compared to the starting cells. The majority of these genes are common to control and induced cells and presumably reflect their common response to starvation. However, a group of 157 genes changes significantly in both aspidocyte samples compared to their direct control, thus perhaps defining a core group of aspidocyte-regulated genes. This is illustrated by the dendrogram based on genewise Spearman correlations amongst the control and aspidocyte samples (Fig. 3B[Fig f3]).

**Fig. 4. f4:**
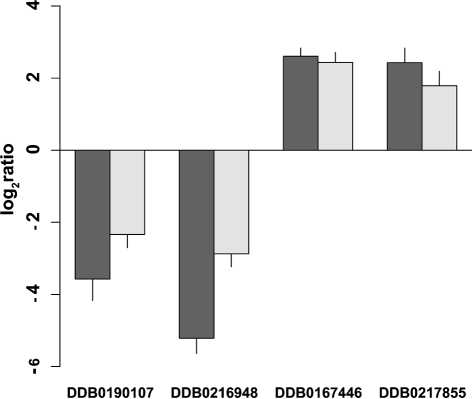
Comparison of RT-PCR and microarray data for four selected genes. Barplot of log_2_ ratios comparing Cd^2+^+DES-treated cells with controls as determined by RT-PCR (dark grey) and microarray (light grey). Error bars display the standard errors of the normalized log ratios. All genes showed significant differences in expression between treated and control (*P*<0.05) according to either a Welch's two-sample *t* test for the RT-PCR data or the Benjamini–Hochberg adjusted *P*-values from the Limma eBayes analysis for the array data.

The genes differentially expressed in aspidocytes (*P*<0.05) compared to control cells are shown in the heat map (Fig. 3C[Fig f3]) and listed in Supplementary Tables S1 and S2, available with the online version of this paper. These results were validated by RT-PCR in which two up-regulated and two down-regulated genes were compared using a group of three unregulated genes as the internal standard (Fig. 4). The ranking of the PCR results completely supports the trends apparent in the microarray data, which if anything appear to underestimate the magnitude of the changes; the overall (Pearson) correlation between the two sets of log_2_ ratios is 0.993.

The top 20 core genes significantly up-regulated in aspidocytes by CdCl_2_/DES and by thiabendazole are listed in Table 4 (for a more extensive list see Supplementary Table S1). They include a number whose predicted function is in lipid metabolism, including fatty acid synthase, phosphatidic acid phosphatase and an MBOAT acyltransferase, all suggesting possible membrane modification in aspidocytes. There is more overlap in the genes down-regulated in the two induction conditions (Supplementary Table S2), but their varied nature is less informative as to how aspidocyte metabolism differs from control cells.

### Mechanism of detergent resistance of aspidocytes

**Fig. 5. f5:**
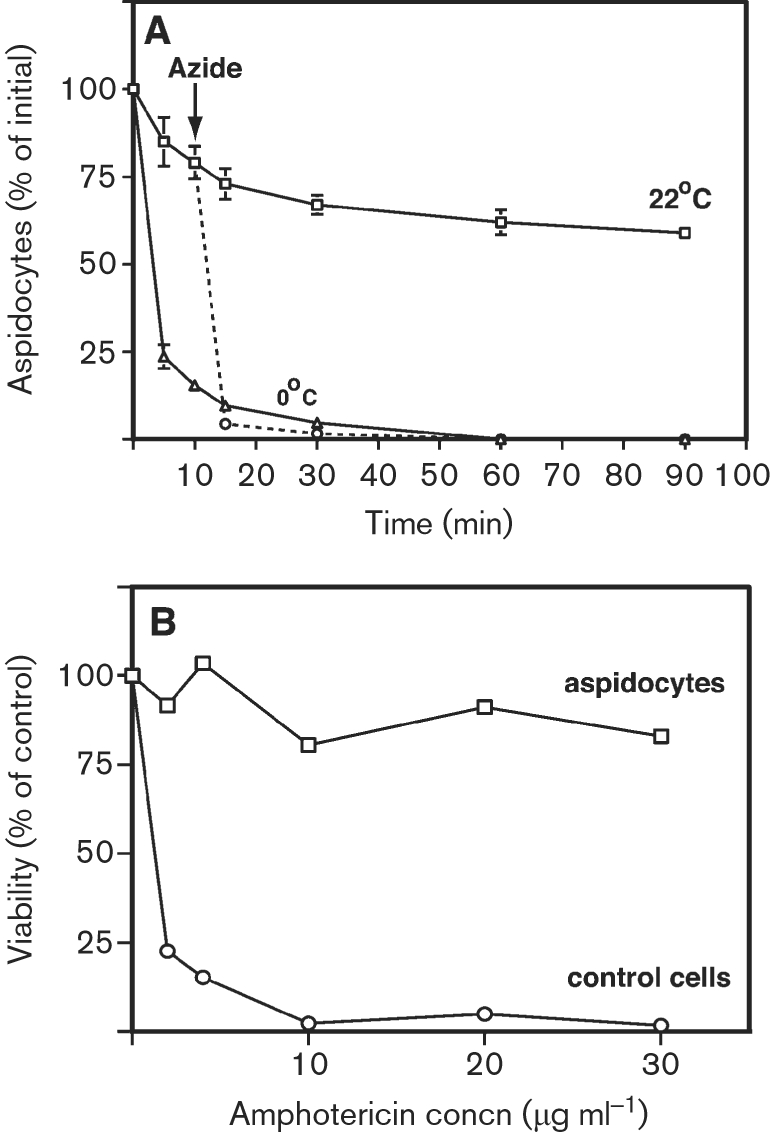
Detergent and amphotericin resistance of aspidocytes. (A) Aspidocyte lysis by detergent. Aspidocytes were incubated in 0.3 % Cemulsol NP12 detergent either at 22 °C, with addition of 2 mM NaN_3_ as indicated, or on ice. Unlysed cells were counted by phase-contrast microscopy at the times indicated. (B) Survival curves in amphotericin B. Aspidocytes or control cells were incubated with the indicated concentrations of amphotericin B for 60 min, and then survivors estimated by clonal dilution onto bacterial lawns and subsequent plaque counting. Aspidocytes were induced in the standard conditions with strain HM1158 for approximately 18 h; results are means of three experiments.

The immediately striking feature of aspidocytes is their resistance to lysis by non-ionic detergents at concentrations that rapidly lyse amoebae. This resistance depends on the presence of 1–2 mM Mg^2+^ in the medium (not shown) and is not absolute, as aspidocytes, unlike spores, progressively lyse over a few hours in 0.3 % Cemulsol NP12 (Fig. 5A[Fig f5]). Aspidocytes are also more resistant than control amoebae to other non-ionic detergents such as Triton X-100 or Tween 20, but again they lyse over time.

One possible explanation for this detergent resistance would be if aspidocytes were able to form a cellulosic cell wall, similar to spores. However, staining for cellulose with Calcofluor showed that aspidocytes lack a cell wall (not shown) and this was confirmed by transmission electron microscopy. Under the electron microscope the plasma membrane of aspidocytes appears identical to that of control cells and the only ultrastructural difference noted was in the mitochondria, which tended to have more distinct cristae (not shown).

The cortical layer of F-actin adds mechanical strength to the plasma membrane, and we wondered whether modification of the actin cytoskeleton might be a feature of aspidocytes, possibly contributing to their detergent resistance. The F-actin cytoskeleton was visualized using a GFP fusion protein that specifically binds F-actin (Pang *et al.*, 1998). Most aspidocytes have a continuous cortex of F-actin superficially similar to control cells. However, about 10 % have a unique double layer of F-actin under the plasma membrane, which we have never observed in control cells (Fig. 1C[Fig f1]).

Having eliminated the possibility of a cell wall, we considered the possibility that aspidocyte detergent resistance might be an active process. To test this idea we used azide or cooling to 0 °C to block ATP production. In both cases, detergent rapidly lyses preformed aspidocytes (Fig. 5A[Fig f5]), showing that their resistance is an active process. Control experiments show that azide alone does not lyse amoebae (Patterson & Spudich, 1995) or aspidocytes (not shown) in short incubations.

Resistance may also be linked to a complete cessation of endocytosis, which can be demonstrated using the membrane-partitioning dye FM1-43 (Aguado-Velasco & Bretscher, 1999). This rapidly stains the plasma membrane of aspidocytes and control cells, and is subsequently internalized in control cells by endocytosis to stain internal membranes. In aspidocytes there is no detectable internal staining even after 20 min incubation, showing that endocytosis is completely blocked (Fig. 1D[Fig f1]). Similar results were obtained with the related dye FM4-64 (not shown).

### Antibiotic resistance

Our working hypothesis is that the function of aspidocytes is to resist levels of environmental toxins that would normally overwhelm amoebae. If this idea is correct, then pre-formed aspidocytes should be able to survive an antibiotic treatment that would kill amoebae. We tested this idea using two toxins, which in preliminary experiments could kill *Dictyostelium* amoebae within 1 h (most of the toxins tested, such as cycloheximide, thiabendazole and daunomycin had little effect on cell viability in this time). Fig. 5B[Fig f5] shows that aspidocytes are strongly resistant to killing by amphotericin B, a membrane-disrupting fungicide produced by streptomycetes (Hartsel & Bolard, 1996), whereas control cells are sensitive. In contrast, their resistance to bleocin, another streptomycete toxin, but one that damages DNA, is only modestly greater than control cells (not shown). Preformed aspidocytes therefore appear to be strongly resistant to at least one class of antibiotics.

## DISCUSSION

Here we report the discovery of what appears to be a new, resistant cell type in *Dictyostelium*. Aspidocytes clearly differ from spores, stalk cells, macrocysts and microcysts in morphology and in their lack of a cellulosic cell wall. They differ from amoebae in their striking detergent resistance and their lack of movement and endocytosis, and they differ from cells undergoing non-vacuolar cell death in their prolonged viability (Huang *et al.*, 2006; Kosta *et al.*, 2004). Aspidocyte formation from amoebae requires protein synthesis and results in a distinct pattern of gene expression, all suggestive of a distinct cell type or state. Once induced, aspidocytes persist for hours without apparent change, but it should also be emphasized that, like the prestalk and prespore cells formed in development, aspidocyte formation is fully reversible: if the inducers are removed, the cells rapidly resume movement and are fully viable.

It is not surprising that aspidocytes have escaped detection in more than 50 years of laboratory study of *Dictyostelium*, given the unusual circumstances required for their initial discovery: a hypersensitive mutant, induction with Cd^2+^ ions and detection by detergent resistance. However, in appropriate conditions aspidocytes can be readily induced in wild-type *D. discoideum* and related social amoebae.

The detergent resistance of aspidocytes takes at least 1 h to appear, but is not due to the synthesis of a cell wall detectable by light or electron microscopy. It is likely that the lipid or protein composition of the plasma membrane is altered, and also that the cessation of endocytosis in aspidocytes increases their resistance to detergent by denying it access to the internal membranes of the endocytic pathway. However, a key feature of the detergent resistance is that it is not merely passive, but requires continued energy metabolism. The nature of this active process can only be speculated upon: one possibility is that aspidocytes are able to expel detergent molecules from their plasma membrane; another is that they somehow repair the lesions in the membrane caused by detergent.

Aspidocyte formation is most efficient from starving cells and is stimulated by a wide variety of agents, including heavy metals, such as cadmium, inhibitors of sterol, protein or RNA synthesis and of mitosis, and also the endogenous DIF signals and their partial mimics, DES, zearalenone (a phyto-oestrogen) and miconazole (Gross *et al.*, 1983; Wang *et al.*, 1990). In most cases, higher concentrations of the inducer are directly toxic to the cell, or at least block aspidocyte formation – thus for instance a high concentration of the protein synthesis inhibitor cycloheximide completely prevents aspidocyte formation.

DIF-1 at roughly physiological concentrations is a poor inducer of aspidocytes, but can synergize effectively with other inducers. This is surprising at first sight, since DIF-1 was discovered as a signal inducing stalk cell differentiation in later development (Morris *et al.*, 1987). However, there is also evidence that DIF-1 can affect cells that have been starved for only a few hours, as in the aspidocyte inductions (Wurster & Kay, 1990), most notably by causing the rapid tyrosine phosphorylation and nuclear localization of STATc (Fukuzawa *et al.*, 2001). This in turn may link DIF-1 into a stress response pathway, since STATc also mediates osmotic and other stress responses in these cells (Araki *et al.*, 2003). Alternatively, DIF-1 may itself be causing mild cellular stress, as it is known to be a mitochondrial uncoupler at unphysiologically high concentrations (Shaulsky & Loomis, 1995).

This synergistic effect of DIF-1 and the wide variety of aspidocyte inducers suggests that several distinct stress pathways must have their inputs integrated to stimulate aspidocyte formation. An important clue as to the identity of one of these pathways comes from the gene disrupted in the hypersensitive mutant. This encodes the metabolic enzyme AMP deaminase, whose activity is one way of removing AMP from the cytoplasm. Activity of this enzyme increases strongly in early development (Jahngen & Rossomando, 1986) and *amdA* mutant cells contain no detectable AMP deaminase activity, resulting in higher AMP levels during starvation than in wild-type cells (Chae *et al.*, 2002). This would in turn be expected to activate AMP-activated protein kinase, which is a known transducer of metabolic stress (Hardie & Hawley, 2001), and therefore a candidate for mediating aspidocyte induction

What is known about aspidocytes suggests that they are an adaptation to resist environmental toxins and antibiotics. They are induced by a wide range of such agents, are formed from separate cells either growing or starving, and are rendered resistant to attack on the plasma membrane by antibiotics such as amphotericin by some unknown mechanism. Once the toxic stress has passed, aspidocytes rapidly lose their resistance and resume amoeboid movement. Since aspidocyte formation overrides development into a fruiting body and is much quicker, aspidocytes seem best adapted to resist a sudden flux of toxins. Even if only a small proportion of the population were induced to become aspidocytes – as is the case with wild-type amoebae in our best current conditions – this would still confer a survival advantage compared to a strain that could produce none. Although *Dictyostelium* is strictly non-pathogenic, other free-living amoebae are opportunistic pathogens (Schuster & Visvesvara, 2004), and it will be interesting to discover whether any of them use a similar strategy to resist drug treatment.

## Figures and Tables

**Table 1. t1:** Aspidocyte induction conditions for the AMP-deaminase (*amdA*) mutant, HM1158, and the wild-type Ax2 Cells were washed free of growth medium and incubated in tissue culture dishes at 10^6^ ml^−1^ in complete medium (10 mM MES pH 6.2, 20 mM KCl, 20 mM NaCl, 1 mM CaCl_2_, 1mM MgCl_2_, 25 μM CdCl_2_, 5 mM cAMP, 100 nM DIF-1) or with CdCl_2_, cAMP or DIF-1 omitted as indicated. After 12–18 h 0.3 % Cemulsol NP12 detergent was added and surviving cells estimated after at least 20 min, either by phase-contrast microscopy (visible) or by clonal dilution onto bacterial lawns and subsequent plaque counting (viable). Results are means±se from four independent experiments.

**Medium**	**Aspidocyte formation (% of total)**			
	***amdA***		**Ax2 (wild-type)**	
	**Visible**	**Viable**	**Visible**	**Viable**
Complete	74.3±6.4	67.8±3.8	9.8±5.6	8.8±4.3
−Cadmium	0	0.16±0.04	0	0.06±0.01
−cAMP	69.7±7.6	65.4±5.5	9.4±4.8	8.1±3.7
−DIF-1	13.2±4.1	7.2±1.0	0	1.27±0.15
−All three	0	0.44±0.12	0	0.12±0.03

**Table 2. t2:** Aspidocyte inducers and their synergy with DIF-1 and CdCl_2_ Cells of the AMP-deaminase (*amdA*) mutant strain HM1158 were washed free of growth medium and incubated in tissue culture dishes at 10^6^ ml^−1^ in a medium containing 10 mM MES pH 6.2, 20 mM KCl, 20 mM NaCl, 1 mM CaCl_2_, 1 mM MgCl_2_ with 2 mM cAMP and the indicated inducers added after 1 h. After 12–18 h, 0.2 % Cemulsol NP12 detergent was added and surviving cells estimated by phase-contrast microscopy after 4–30 min exposure to detergent. All agents were initially tested over a range of concentrations, with the most effective one given. Results with errors (se) are from three to eight independent experiments and those without from two experiments; they are given as a percentage of the input cells, which it should be noted can divide to a limited extent (control cell numbers increased by approximately 70 % in the incubation).

**Compound**	**Concn (μM)**	**Aspidocytes (% of input cells)**		
		**None**	**DIF-1 (100 nM)**	**CdCl_2_ (25 μM)**
None	–	0.2±0.3	0.5±0.6	1.3±1.3
CdCl_2_	25	2.4±1.8	66.1±27.2	–
CdCl_2_	200	64.5±32.1	84.0±30.7	–
HgCl_2_	5	1.1±0.8	8.9±7.8	–
Thiabendazole	50	0.3±0.3	2.6±0.9	18.8±12.7
Thiabendazole	300	55.9±27.8	91.4±22.4	85.8±34.9
Miconazole	10	121±16.3	111±19.8	118±10.4
Clotrimazole	10	61.3±22.2	81.1±21.0	95.9
Econazole	10	130±25.9	110±21.1	116
Cycloheximide	710	1.2±1.1	44.1±23.9	2.8±3.0
Actinomycin D	10	5.6±3.2	27.3±13.8	5.3±3.3
G418	4	0.2±0.4	5.2±4.9	4.5±5.9
DES	10	53.5±9.4	51.0±21.6	92.3±11.1
Zearalenone	50	18.6±7.6	37.9±16.6	70.7±16.2

**Table 3. t3:** Synergistic induction of aspidocytes by DIF analogues in the presence of cadmium Cells of the AMP-deaminase (*amdA*) mutant strain HM1158 were washed free of growth medium and incubated in tissue culture dishes at 10^6^ ml^−1^ in medium containing 10 mM MES pH 6.2, 20 mM KCl, 20 mM NaCl, 1 mM CaCl_2_, 1 mM MgCl_2_, 5 mM cAMP, 25 μM CdCl_2_ with the indicated additions. After 12–18 h, 0.3 % Cemulsol NP12 detergent was added and surviving cells estimated by phase-contrast microscopy after at least 20 min exposure to detergent; results were confirmed by clonal dilution of cells onto bacterial lawns and subsequent plaque counting (not shown). Results are means±se from three independent experiments and are normalized to +DIF-1, which gave 76 % aspidocytes. DIF-1 [1-(3,5-dichloro-2,6-dihydroxy-4-methoxyphenyl)-1-hexanone] is an endogenous stalk-cell inducing signal; DIF-2 the pentanone homologue produced in smaller amounts; DIF-3 the first breakdown product of DIF-1; THPH, Cl-THPH and dM-DIF-1 are successive biosynthetic intermediates (Kay, 1998).

**Addition (100 nM)**	**Aspidocyte formation (% of DIF-1 response)**	Relative bioactivity*
None	11.6	–
DIF-1	100	100
DIF-2	86.3 (±7.6)	40.6
DIF-3	82.8 (±6.3)	3.5
THPH	5.8 (±1.6)	0.00002
Cl-THPH	8.8 (±1.8)	nd
dM-DIF-1	16.2 (±2.1)	0.4

*Bioactivities from Insall & Kay (1990) and Masento *et al*. (1988) determined using an assay for stalk cell formation where 10^−10^ M DIF-1 gives approximately 50 % response. nd, Not done.

**Table 4. t4:** Genes up-regulated in aspidocytes Aspidocytes were induced for 6 h in suspension from starving HM1158 cells by either 250 μM thiabendazole or 25 μM CdCl_2_ plus 10 μM DES and gene expression relative to controls determined using micro-arrays. Two independent replicates were carried out for the thiabendazole and five for the CdCl_2_/DES induction. The top 20 genes significantly induced (*P*<0.05) in both conditions are annotated and ranked by their induction with CdCl_2_/DES. Gene IDs are from Dictybase (http://dictybase.org/). Inductions compared to control are given as log_2_; homology was recognized by Pfam Hidden Markov models in the annotated genome at GeneDB (http://www.genedb.org/genedb/dicty/) and blast searches; likely function is given where there is a clear indication from homology.

**Gene**	**Fold increase (log_2_)**		**Homology**	**Possible function**
	**Cd/DES**	**Thiab**		
DDB0167446	2.44	1.71	Fatty acid synthase	Lipid metabolism
DDB0217855	1.79	1.79	Calcineurin	
DDB0187927	1.32	0.727	TBC domain	
DDB0190208	1.29	1.38	*stlA* steely PKS	Polyketide synthesis
DDB0206002	1.20	2.28	Carboxylesterase	
DDB0190313	1.13	0.922	Pyridoxal enzyme	Cys/Met metabolism
DDB0205486	1.12	1.19	Cdc2 protein kinase	Cell cycle
DDB0191696	0.963	0.837	Mannosidase	
DDB0189935	0.821	0.519	Aminotransferase	Amino acid metabolism
DDB0203373	0.781	0.787	Glyoxalase	
DDB0217225	0.776	1.38	Hsp70	Stress response
DDB0186419	0.736	0.810	Saccharopine deH	Amino acid metabolism
DDB0190518	0.731	0.690	CDP-DAG synthase	Lipid metabolism
DDB0204255	0.726	0.812	Alcohol dehydrogenase	
DDB0167402	0.674	0.626	Steroid dehydrogenase?	Lipid metabolism?
DDB0169135	0.667	1.18	Pyridoxal enzyme	Amino acid metabolism
DDB0205259	0.660	1.35	MBOAT acyltransferase	Lipid metabolism
DDB0202283	0.656	0.900	PAP2 phosphatase	Lipid metabolism
DDB0205299	0.646	1.13	rRNA methyltransferase	Stress response
DDB0217052	0.622	1.17	None	
DDB0168546	0.601	0.900	Cytidine deaminase	
DDB0187085	0.583	0.942	Acyl-CoA oxidase	Lipid metabolism
DDB0168479	0.528	1.27	PEP carboxykinase	Carbohydrate metabolism
DDB0183812	0.393	1.27	Importin beta	Nuclear uptake
DDB0190878	0.374	0.773	*hspB* Hsp70 cognate	Stress response

## Abbreviations

- DIF, differentiation inducing factor
- DES, diethylstilboestrol

## References

[r1] Aguado-Velasco, C. & Bretscher, M. S. (1999). Circulation of the plasma membrane in *Dictyostelium*. Mol Biol Cell 10, 4419–4427. 10588667 10.1091/mbc.10.12.4419 PMC25767

[r2] Araki, T., Tsujioka, M., Abe, T., Fukuzawa, M., Meima, M., Schaap, P., Morio, T., Urushihara, H., Katoh, M. & other authors (2003). A STAT-regulated, stress-induced signalling pathway in *Dictyostelium*. J Cell Sci 116, 2907–2915. 12771188 10.1242/jcs.00501

[r3] Austin, M. B., Saito, T., Bowman, M. E., Haydock, S., Kato, A., Moore, B. S., Kay, R. R. & Noel, J. P. (2006). Biosynthesis of *Dictyostelium* Differentiation Inducing Factor by a hybrid type I fatty acid-type III polyketide synthase. Nat Chem Biol 2, 494–502. 16906151 10.1038/nchembio811 PMC2864586

[r4] Balaban, N. Q., Merrin, J., Chait, R., Kowalik, L. & Leibler, S. (2004). Bacterial persistence as a phenotypic switch. Science 305, 1622–1625. 15308767 10.1126/science.1099390

[r5] Benjamini, Y. & Hochberg, Y. (1995). Controlling the false discovery rate: a practical and powerful approach to multiple testing. J R Stat Soc Ser B 57, 289–300.

[r6] Burlando, B., Evangelisti, V., Dondero, F., Pons, G., Camakaris, J. & Viarengo, A. (2002). Occurrence of Cu-ATPase in *Dictyostelium*: possible role in resistance to copper. Biochem Biophys Res Commun 291, 476–483. 11855813 10.1006/bbrc.2002.6463

[r7] Chae, S. C., Fuller, D. & Loomis, W. F. (2002). Altered cell-type proportioning in *Dictyostelium* lacking adenosine monophosphate deaminase. Dev Biol 241, 183–194. 11784104 10.1006/dbio.2001.0491

[r8] Eichinger, L., Pachebat, J. A., Glockner, G. & other authors (2005). The genome of the social amoeba *Dictyostelium discoideum*. Nature 435, 43–57. 15875012 10.1038/nature03481 PMC1352341

[r9] Errington, J. (2003). Regulation of endospore formation in *Bacillus subtilis*. Nat Rev Microbiol 1, 117–126. 15035041 10.1038/nrmicro750

[r10] Felsenstein, J. (2005). phylip – Phylogeny Inference Package, version 3.65. Distributed by the author. University of Washington, Seattle, USA.

[r11] Firtel, R., Baxter, L. & Lodish, H. (1973). Actinomycin D and the regulation of enzyme biosynthesis during development of *Dictyostelium discoideum*. J Mol Biol 79, 315–327. 4796770 10.1016/0022-2836(73)90008-9

[r12] Fukuzawa, M., Araki, T., Adrian, I. & Williams, J. G. (2001). Tyrosine phosphorylation-independent nuclear translocation of a *Dictyostelium* STAT in response to DIF signaling. Mol Cell 7, 779–788. 11336701 10.1016/s1097-2765(01)00222-2

[r13] Gentleman, R. C., Carey, V. J., Bates, D. M. & other authors (2004). Bioconductor: open software development for computational biology and bioinformatics. Genome Biol 5, R80. 15461798 10.1186/gb-2004-5-10-r80 PMC545600

[r14] Gross, J. D., Bradbury, J., Kay, R. R. & Peacey, M. J. (1983). Intracellular pH and the control of cell differentiation in *Dictyostelium discoideum*. Nature 303, 244–245. 6843673 10.1038/303244a0

[r15] Gross, J. D., Peacey, M. J. & Pogge von Strandmann, R. (1988). Plasma membrane proton pump inhibition and stalk cell differentiation in *Dictyostelium discoideum*. Differentiation 38, 91–98. 2850252 10.1111/j.1432-0436.1988.tb00202.x

[r16] Hall-Stoodley, L., Costerton, J. W. & Stoodley, P. (2004). Bacterial biofilms: from the natural environment to infectious diseases. Nat Rev Microbiol 2, 95–108. 15040259 10.1038/nrmicro821

[r17] Hardie, D. G. & Hawley, S. A. (2001). AMP-activated protein kinase: the energy charge hypothesis revisited. Bioessays 23, 1112–1119. 11746230 10.1002/bies.10009

[r18] Hartsel, S. & Bolard, J. (1996). Amphotericin B: new life for an old drug. Trends Pharmacol Sci 17, 445–449. 9014498 10.1016/s0165-6147(96)01012-7

[r19] Hoffman, L. R., D'Argenio, D. A., MacCoss, M. J., Zhang, Z., Jones, R. A. & Miller, S. I. (2005). Aminoglycoside antibiotics induce bacterial biofilm formation. Nature 436, 1171–1175. 16121184 10.1038/nature03912

[r20] Horn, E. G. (1971). Food competition among the cellular slime molds (Acrasiae). Ecology 52, 475–484.

[r21] Huang, E., Blagg, S. L., Keller, T., Katoh, M., Shaulsky, G. & Thompson, C. R. (2006). bZIP transcription factor interactions regulate DIF responses in *Dictyostelium*. Development 133, 449–458. 16410410 10.1242/dev.02240 PMC3531922

[r22] Huss, M. J. (1989). Dispersal of cellular slime moulds by two soil invertebrates. Mycologia 81, 677–682.

[r23] Insall, R. & Kay, R. R. (1990). A specific DIF binding protein in *Dictyostelium*. EMBO J 9, 3323–3328. 2170113 10.1002/j.1460-2075.1990.tb07532.x PMC552069

[r24] Jahngen, E. G. E. & Rossomando, E. F. (1986). AMP deaminase in *Dictyostelium discoideum*: increase in activity following nutrient deprivation induced by starvation or hadacidin. Mol Cell Biochem 71, 71–78. 3014311 10.1007/BF00219330

[r25] Kay, R. R. (1987). Cell differentiation in monolayers and the investigation of slime mold morphogens. Methods Cell Biol 28, 433–448. 3600415 10.1016/s0091-679x(08)61661-1

[r26] Kay, R. R. (1989). Evidence that elevated intracellular cyclic AMP triggers spore maturation in *Dictyostelium*. Development 105, 753–759.

[r27] Kay, R. R. (1998). The biosynthesis of differentiation-inducing factor, a chlorinated signal molecule regulating *Dictyostelium* development. J Biol Chem 273, 2669–2675. 9446571 10.1074/jbc.273.5.2669

[r28] Kay, R. R., Gadian, D. G. & Williams, S. R. (1986). Intracellular pH in *Dictyostelium*: a ^31^P nuclear magnetic resonance study of its regulation and possible role in controlling cell differentiation. J Cell Sci 83, 165–179. 3805139 10.1242/jcs.83.1.165

[r29] Keim, M., Williams, R. S. & Harwood, A. J. (2004). An inverse PCR technique to rapidly isolate the flanking DNA of Dictyostelium insertion mutants. Mol Biotechnol 26, 221–224. 15004291 10.1385/MB:26:3:221

[r30] Kessin, R. H. (2001). *Dictyostelium*. Cambridge: Cambridge University Press.

[r31] Ketcham, R. B., Levitan, D. R., Shenk, M. A. & Eisenberg, R. M. (1988). Do interactions of cellular slime mold species regulate their densities in soil? Ecology 69, 193–199.

[r32] Kooperberg, C., Fazzio, T. G., Delrow, J. J. & Tsukiyama, T. (2002). Improved background correction for spotted DNA microarrays. J Comput Biol 9, 55–66. 11911795 10.1089/10665270252833190

[r33] Kosta, A., Roisin-Bouffay, C., Luciani, M. F., Otto, G. P., Kessin, R. H. & Golstein, P. (2004). Autophagy gene disruption reveals a non-vacuolar cell death pathway in *Dictyostelium*. J Biol Chem 279, 48404–48409. 15358773 10.1074/jbc.M408924200

[r34] Kuspa, A. & Loomis, W. F. (1992). Tagging developmental genes in *Dictyostelium* by restriction enzyme-mediated integration of plasmid DNA. Proc Natl Acad Sci U S A 89, 8803–8807. 1326764 10.1073/pnas.89.18.8803 PMC50009

[r35] Lupetti, A., Danesi, R., Campa, M., Del Tacca, M. & Kelly, S. (2002). Molecular basis of resistance to azole antifungals. Trends Mol Med 8, 76–81. 11815273 10.1016/s1471-4914(02)02280-3

[r36] Masento, M. S., Morris, H. R., Taylor, G. W., Johnson, S. J., Skapski, A. C. & Kay, R. R. (1988). Differentiation-inducing factor from the slime mould *Dictyostelium discoideum* and its analogues. Biochem J 256, 23–28. 3223901 10.1042/bj2560023 PMC1135362

[r37] Mizukami, Y. & Iwabuchi, M. (1970). Effects of actinomycin D and cycloheximide on the morphogenesis and synthesis of RNA and protein in the cellular slime mold, *Dictyostelium discoideum*. Exp Cell Res 63, 317–324. 5530917 10.1016/0014-4827(70)90219-3

[r38] Morris, H. R., Taylor, G. W., Masento, M. S., Jermyn, K. A. & Kay, R. R. (1987). Chemical structure of the morphogen differentiation inducing factor from *Dictyostelium discoideum*. Nature 328, 811–814. 3627228 10.1038/328811a0

[r39] Nellen, W., Silan, C. & Firtel, R. A. (1984). DNA-mediated transformation in *Dictyostelium discoideum*: regulated expression of an actin gene fusion. Mol Cell Biol 4, 2890–2898. 6098825 10.1128/mcb.4.12.2890-2898.1984 PMC369302

[r40] Pang, K. M., Lee, E. & Knecht, D. A. (1998). Use of a fusion protein between GFP and an actin-binding domain to visualize transient filamentous-actin structures. Curr Biol 8, 405–408. 9545201 10.1016/s0960-9822(98)70159-9

[r41] Patterson, B. & Spudich, J. A. (1995). A novel positive selection for identifying cold-sensitive myosin II mutants in *Dictyostelium*. Genetics 140, 505–515. 7498732 10.1093/genetics/140.2.505 PMC1206630

[r42] Pogge-von Strandmann, R., Kay, R. R. & Dufour, J.-P. (1984). An electrogenic proton pump in plasma membranes from the cellular slime mould *Dictyostelium discoideum*. FEBS Lett 175, 422–427.

[r43] Raper, K. B. (1984). *The Dictyostelids*. Princeton, NJ: Princeton University Press.

[r44] Schuster, F. L. & Visvesvara, G. S. (2004). Free-living amoebae as opportunistic and non-opportunistic pathogens of humans and animals. Int J Parasitol 34, 1001–1027. 15313128 10.1016/j.ijpara.2004.06.004

[r45] Serafimidis, I. & Kay, R. R. (2005). New prestalk and prespore inducing signals in *Dictyostelium*. Dev Biol 282, 432–441. 15950608 10.1016/j.ydbio.2005.03.023

[r46] Shaulsky, G. & Loomis, W. F. (1995). Mitochondrial DNA replication but no nuclear DNA replication during development of *Dictyostelium*. Proc Natl Acad Sci U S A 92, 5660–5663. 7777565 10.1073/pnas.92.12.5660 PMC41756

[r47] Smyth, G. K. (2004). Linear models and empirical Bayes methods for assessing differential expression in microarray experiments.Stat Appl Genet Mol Biol 3, Article 3. 10.2202/1544-6115.1027 16646809

[r48] Smyth, G. K. (2005). Limma: linear models for microarray data. In *Bioinformatics and Computational Biology Solutions using R and Bioconductor*. Edited by V. C. R. Gentleman, S. Dudoit, R. Irizarry & W. Huber. New York: Springer.

[r49] Thompson, C. R., Fu, Q., Buhay, C., Kay, R. R. & Shaulsky, G. (2004). A bZIP/bRLZ transcription factor required for DIF signaling in *Dictyostelium*. Development 131, 513–523. 14729573 10.1242/dev.00939

[r50] Wallace, M. A. & Raper, K. B. (1979). Genetic exchanges in the macrocysts of *Dictyostelium discoideum*. J Gen Microbiol 113, 327–337. 292755 10.1099/00221287-113-2-327

[r51] Wang, M., Roelfsema, J. H., Williams, J. G. & Schaap, P. (1990). Cytoplasmic acidification facilitates but does not mediate DIF-induced prestalk gene expression in *Dictyostelium discoideum*. Dev Biol 140, 182–188. 2162791 10.1016/0012-1606(90)90065-q

[r52] Watts, D. J. & Ashworth, J. M. (1970). Growth of myxamoebae of the cellular slime mould *Dictyostelium discoideum* in axenic culture. Biochem J 119, 171–174. 5530748 10.1042/bj1190171 PMC1179339

[r53] Welker, D. L. & Williams, K. L. (1980). Mitotic arrest and chromosome doubling using thiabendazole, cambendazole, nocodazole, and benlate in the slime mould *Dictyostelium discoideum*. J Gen Microbiol 116, 397–407.

[r54] Wurster, B. & Kay, R. R. (1990). New roles for DIF? Effects on early development in *Dictyostelium*. Dev Biol 140, 189–195. 2162792 10.1016/0012-1606(90)90066-r

[r55] Zischka, H., Oehme, F., Pintsch, T., Ott, A., Keller, H., Kellermann, J. & Schuster, S. C. (1999). Rearrangement of cortex proteins constitutes an osmoprotective mechanism in *Dictyostelium*. EMBO J 18, 4241–4249. 10428962 10.1093/emboj/18.15.4241 PMC1171500

